# Analytical Performance of the FreeStyle Libre 2 Glucose Sensor in Healthy Male Adults

**DOI:** 10.3390/s24175769

**Published:** 2024-09-05

**Authors:** Eva Fellinger, Tom Brandt, Justin Creutzburg, Tessa Rommerskirchen, Annette Schmidt

**Affiliations:** 1 NextGenerationEU, dtec.bw Project Smart Health Lab, University of the Bundeswehr Munich, 85579 Neubiberg, Germany; 2 Institute of Sport Sciences, University of the Bundeswehr Munich, 85579 Neubiberg, Germany; tom.brandt@unibw.de (T.B.);; 3 Research Center Smart Digital Health, University of the Bundeswehr Munich, 85579 Neubiberg, Germany

**Keywords:** CGM, glycemic control, MARD, oral glucose tolerance test, sensor accuracy

## Abstract

Continuous Glucose Monitoring (CGM) not only can be used for glycemic control in chronic diseases (e.g., diabetes), but is increasingly being utilized by individuals and athletes to monitor fluctuations in training and everyday life. However, it is not clear how accurately CGM reflects plasma glucose concentration in a healthy population in the absence of chronic diseases. In an oral glucose tolerance test (OGTT) with forty-four healthy male subjects (25.5 ± 4.5 years), the interstitial fluid glucose (ISFG) concentration obtained by a CGM sensor was compared against finger-prick capillary plasma glucose (CPG) concentration at fasting baseline (T0) and 30 (T30), 60 (T60), 90 (T90), and 120 (T120) min post OGTT to investigate differences in measurement accuracy. The overall mean absolute relative difference (MARD) was 12.9% (95%-CI: 11.8–14.0%). Approximately 100% of the ISFG values were within zones A and B in the Consensus Error Grid, indicating clinical accuracy. A paired t-test revealed statistically significant differences between CPG and ISFG at all time points (T0: 97.3 mg/dL vs. 89.7 mg/dL, T30: 159.9 mg/dL vs. 144.3 mg/dL, T60: 134.8 mg/dL vs. 126.2 mg/dL, T90: 113.7 mg/dL vs. 99.3 mg/dL, and T120: 91.8 mg/dL vs. 82.6 mg/dL; *p* < 0.001) with medium to large effect sizes (d = 0.57–1.02) and with ISFG systematically under-reporting the reference system CPG. CGM sensors provide a convenient and reliable method for monitoring blood glucose in the everyday lives of healthy adults. Nonetheless, their use in clinical settings wherein implications are drawn from CGM readings should be handled carefully.

## 1. Introduction

In diabetes management, the accurate and reliable measurement of blood glucose levels is crucial for ensuring appropriate therapy (e.g., the right timing for insulin doses) and avoiding complications, such as phases of hypo- and hyperglycemia. Traditionally, blood glucose measurements (also referred to as self-monitoring blood glucose [SMBG]) have been performed using capillary blood, wherein a small amount of blood is drawn onto a test strip and is analyzed by a glucometer. However, in recent years, CGM has gained importance, as it allows for the continuous monitoring of glucose levels without the need for repeated finger sticks [1]. The analytical performance of CGM sensors is an important issue that determines the accuracy and reliability of this technology in clinical practice. A multitude of studies exist wherein the precision, accuracy, and performance of different CGM systems in type 1 and type 2 diabetes are investigated, either in head-to-head comparisons [2] or against one designated comparator method [3,4].

Moreover, beyond diabetic populations, there is growing interest in the application of CGM technology for normoglycemic individuals and athletes wishing to gain insight into their glycemic profiles for daily supervision or performance enhancement [5]. Additionally, more and more studies focus on non-diabetic cohorts to gain insight into the glycemic profiles of healthy individuals [6,7,8]. Continuous monitoring provides valuable insights into the dynamics of glucose metabolism, aiding in the potential optimization of dietary habits, physical activity, and overall health management [9]. Thus, understanding the analytical performance of CGM sensors not only benefits diabetic patients, but also holds potential for enhancing the well-being and health outcomes of normoglycemic individuals.

Nevertheless, there is no analytical performance study for the Abbott Freestyle Libre 2 Sensor (Abbott Diabetes Care, IL), which so far is using healthy, male, non-diabetic subjects, even though similar studies including female and geriatric individuals as study populations and venous blood samples as part of comparator methods exist [10,11]. Therefore, the purpose of this study is to evaluate the measurement accuracy and the analytical performance of interstitial fluid glucose (ISFG) measurements obtained by the Abbott Freestyle Libre 2 sensor (CGM) against capillary blood glucose (CPG) measurements obtained by the finger-prick method in response to an oral glucose tolerance test in healthy male adults.

## 2. Materials and Methods

### 2.1. Trial Oversight

**Figure 1:**
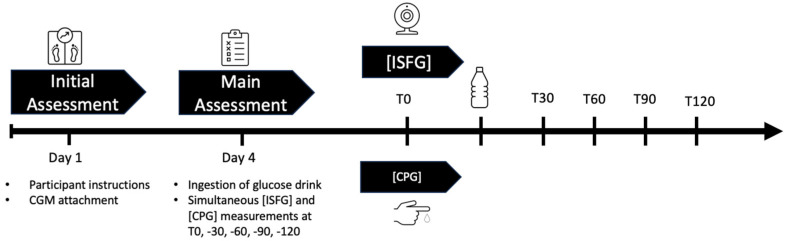
Graphical overview of the study design: On day 1 (initial assessment), participants were instructed and CGM sensors were applied onto their attachment site. On day 4, after a carbohydrate-rich nutrition for the last three days and an 8–12 h fast, they were asked to ingest a glucose drink to start the OGTT. Simultaneous ISFG and CPG were taken at T0, T30, T60, T90, and T120. Abbreviations: CGM = continuous glucose measurement, ISFG = interstitial fluid glucose, CPG = capillary glucose, OGTT = oral glucose tolerance test, T0 = fasting baseline, T30, T60, T90, T120 = 30, 60, 90, 120 min post OGTT.

This study followed an observational, prospective design and was conducted between April 2023 and June 2023 in accordance with the ethical standards of the 1975 Declaration of Helsinki. After an initial assessment wherein sensor placement and participant instruction took place, on the main assessment day, participants had to perform an OGTT, then ISFG and CPG were obtained simultaneously at five different time points: at fasting baseline (T0) and 30 min (T30), 60 min (T60), 90 min (T90), and 120 min (T120) post OGTT. The study took place at the UniBw M in Neubiberg, Germany. Participants were recruited through internal email distribution services, where the aims and procedures of the study were presented. An overview of the study design is displayed in Figure 1.

### 2.2. Participants

Men between the ages of 18 and 40 were invited to participate in the study. Exclusion criteria were chronic diseases like diabetes and the intake of medications (e.g., cortisone) that could impair glucose metabolism. The total sample comprised 68 participants. Considering all exclusion criteria and refusal to participate, this resulted in a final study population of 44 subjects (220 paired glucose measurements). The mean age was 25.49 ± 4.47 years with a mean body mass of 84.94 ± 13.42 kilograms (kg). See Table 1 for descriptive characteristics of the study population and further information about the anthropometric data.

### 2.3. Study Design

#### 2.3.1. Initial Assessment

The initial assessment consisted of the participant instructions and the application of the CGM sensor. After cleaning the skin with an alcohol swab, each participant’s non-dominant upper arm was fitted with a CGM Sensor (FreeStyle Libre 2, Abbott Diabetes Care Inc., UK) in accordance with the manufacturer’s instructions, and it was worn for three days before the study measurements started. The sensor was manufacturer-calibrated and needed no further calibration. The participants were instructed not to take any blood-thinning or other medications (such as aspirin and other painkillers) in the three days prior to the measurement and not to engage in any moderate- to high-intensity physical activity for 24 hours (h) before the laboratory visit. In addition, participants were required to ingest a high-carb diet (150–250 grams [gr] per day) for at least three days and were instructed to fast for 8–12 h prior to the laboratory visit for the main assessment.

#### 2.3.2. Main Assessment

The participants arrived 72 h after the application of the CGM sensor at the laboratory and rested for 5 min before providing a fasting capillary blood sample. Each time a CPG sample was taken, the CGM was scanned manually with a mobile phone serving as the data receiver, equipped with the appropriate application (Freestyle Libre Link 2 App, Abbott Diabetes Care, IL) by the participants themselves to obtain simultaneous ISFG readings from both sites. After that, they were asked to consume a drink of 300 milliliters (mL) containing 82.5 g glucose monohydrate (in line with the standard OGTT procedure) [12]. Subsequent ISFG and CPG samples were collected at T30, T60, T90, and T120 after the glucose drink consumption was initiated.

#### 2.3.3. Blood Sampling and Analysis

For sample collection, the conventional finger-prick method was used to obtain CPG. This method served as the reference method for further comparisons. Therefore, a finger was cleaned with an alcohol swab and then pricked with a lancet (Haemolance, A.F.S.-Biotechnik GmbH, Ludwigsstadt, Germany). For testing, 0.3 to 1.2 microliters (μL) of capillary blood were drawn onto a test strip (Accu-chek, Roche Diabetes Care Deutschland GmbH, Mannheim, Germany) and analyzed by a glucometer (Accu-chek Guide, Roche Diabetes Care Deutschland GmbH, Mannheim, Germany). When the blood contacts the test strip, the glucose is oxidized by the enzyme glucose oxidase, releasing electrons and creating a current. It is measured by an electrode and is directly proportional to the glucose concentration in the blood.

### 2.4. Statistical Approach and CGM Accuracy

ISFG and CPG measurements were paired at five discrete time points: T0, T30, T60, T90, and T120. For the assessment of sensor accuracy, different methods were applied: The mean absolute relative difference and the systematic measurement difference were calculated, ISO 15197:2013 criteria [13] were evaluated, and a paired t-test was used to analyze the difference between timepoints.

#### 2.4.1. Mean Absolute Relative Difference (MARD)

MARD provides a metric value that reflects the overall accuracy of the CGM system. Hereby, the mean absolute relative difference (in percent) between the ISFG and CPG measurements is calculated. Small percentage values indicate high agreement of the CGM system and the reference method (CPG) [14]. The formula utilized to determine MARD was:

MARD = mean (abs ((ISFG − CPG)/CPG)) × 100(1)

#### 2.4.2. ISO 15197:2013 Criteria

The International Organization for Standardization (ISO) 15197:2013 standards, which were primarily created for glucometers, were evaluated to see what proportion of paired findings matched these requirements [15]. According to ISO 15197:2013, two conditions need to be fulfilled to meet the accuracy requirements for glucometers.

1. When the reference CPG is less than 100 mg/dL (resp. 5.56 mmol/L), 95% of the device’s ISFG results must fall within ±15 mg/dL (0.83 mmol/L) of the reference value CPG or within ±15% when the reference glucose values are greater than 100 mg/dL;
2. At least 99% of results must fall within zones A and B in the Consensus Error Grid (CEG), also referred to as the Parkes Error Grid. Zones are defined as follows: zone A (no effect on clinical action), zone B (altered clinical action with little or no effect on clinical outcomes), zone C (altered clinical action and likely to affect clinical outcomes), zone D (altered clinical action that could have significant medical risks), and zone E (altered clinical action that could have dangerous consequences).

#### 2.4.3. Systematic Measurement Difference (Bias)

Furthermore, a Bland–Altman plot was used to analyze the systematic measurement difference of the two different measurement systems [16]. In contrast to MARD, bias takes into account the directionality of the difference—whether it is positive or negative—in relation to the comparison method’s value [17]. Therefore, each individual difference between CPG and ISFG was compared to their individual mean value of the paired glucose measurement. The limits of agreement were calculated as the mean difference ± 1.96 standard deviation of the difference.

#### 2.4.4. Differences between Discrete Timepoints

A paired t-test was conducted to examine the differences between CPG and ISFG at timepoints T0, T30, T60, T90, and T120. The effect sizes of the differences between measurement systems were calculated according to Cohen’s d: small (0.2–0.5), moderate (0.5–0.8), and large (>0.8). Statistical significance was accepted at *p* < 0.05 (two-sided). The data analysis was performed with SPSS 29^®^ (IBM SPSS, Armonk, NY, USA), and graphs were created using R (version 4.3.2) software.

## 3. Results

### 3.1. Mean Absolute Relative Difference (MARD)

The overall MARD was 12.9% (95%-CI: 11.8–14%), with MARDs at each timepoint ranging from 10.9 to 14.6% (Table 2).

### 3.2. ISO 15197:2013 Criteria

**Figure 2:**
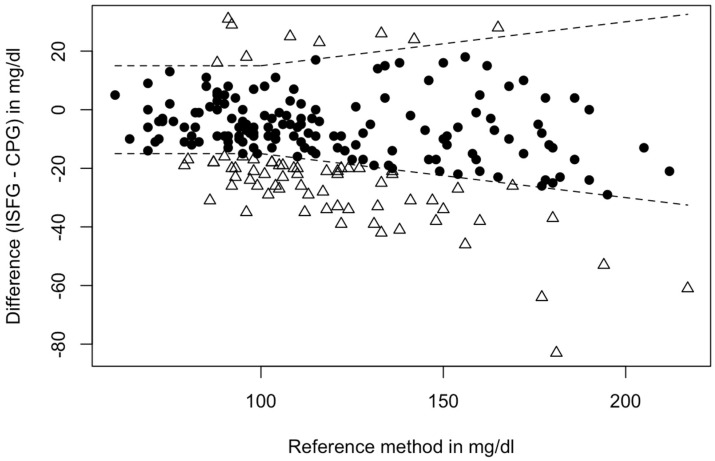
Fulfillment of accuracy criteria: Dots (N = 149) depict matched glucose pairs meeting the ISO 15197:2013 criteria, and triangles (N = 71) lie outside the boundaries of the ISO 15197:2013. Note: Boundaries are as follows: If reference glucose CPG is <100 mg/dL, ISFG must fall within ±15 mg/dL of reference glucose or within ±15% when the reference glucose values are greater than 100 mg/dL. Approximately 95% of matched glucose pairs shall fall within these boundaries to satisfy the ISO 15197:2013 criteria. In this case, 67.7% met the necessary requirements. Abbreviations: CPG = capillary blood glucose, ISFG = interstitial fluid glucose.

In total, 67.7% (n = 149) of the paired glucose data satisfied the first ISO 15197:2013 requirement and were within the permissible range (Figure 2).

**Figure 3:**
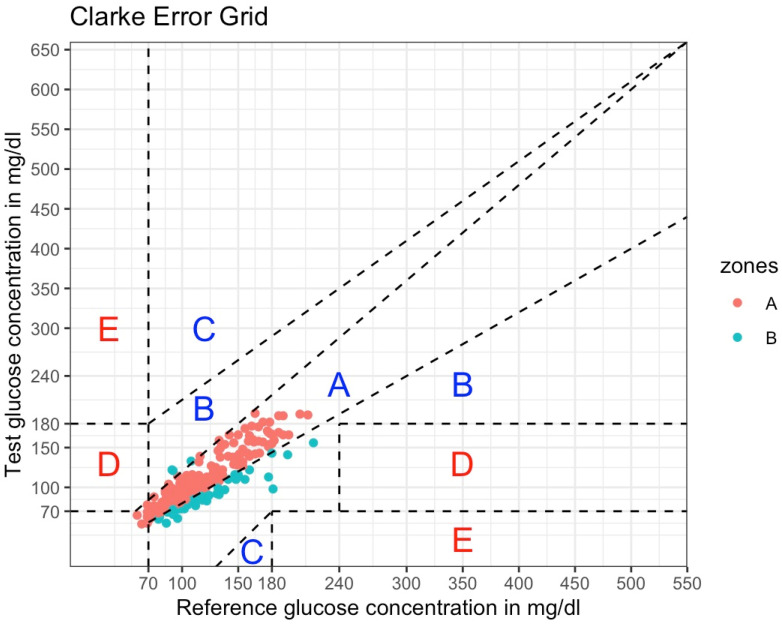
Consensus Error Grid analysis of the Freestyle Libre 2 Sensor (220 matched glucose pairs). ISFG measurements (interstitial fluid glucose; serves as test glucose concentration) were plotted against CPG measurements (capillary glucose; serves as reference glucose concentration). According to ISO 15197:2013, 99% of matched glucose pairs should fall in zones A and B. Dashed lines depict the boundaries of the zones, implying different degrees of risk for clinical decision-making. Red dots are values falling into zone A (no effect on clinical action), whereas blue dots are values falling into zone B (altered clinical action with little or no effect on clinical outcomes). Zone C: altered clinical action that is likely to affect clinical outcomes. Zone D: altered clinical action that could have significant medical risks. Zone E: altered clinical action that could have dangerous consequences.

When examining the clinical accuracy of CGM-derived data in the CEG analysis, 82.7% (n = 182) of the sensor readings were found in zone A and 17.3% (n = 38) in zone B (Figure 3).

### 3.3. Systematic Measurement Difference (Bias)

**Figure 4:**
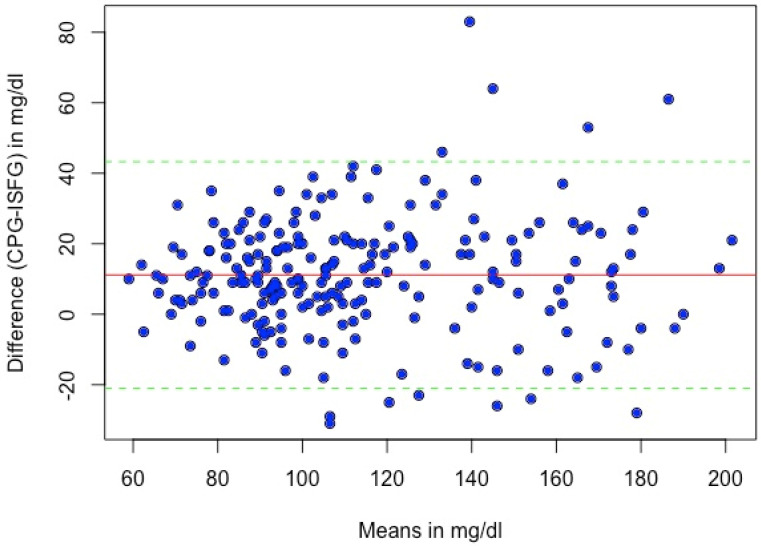
Bland–Altman plot of sensor and capillary glucose levels. The solid red line represents the mean difference between the sensor and capillary glucose values (11.1 mg/dL); the dashed lines indicate 1.96 × SD of the difference. Abbreviations: CPG = capillary blood glucose, ISFG = interstitial fluid glucose.

The systematic bias between CPG and ISFG was 11.1 mg/dL, and the 95% Limits of Agreement (LoA) ranged from −20.7 to 42.8 mg/dL. A Bland–Altman analysis revealed that 5.4% (N = 12) of the matched glucose pairs did not meet the LoA (mean difference ± 1.96*SD of difference). The differences between the individual glucose measurements of the two methods plotted against their mean are presented in Figure 4.

### 3.4. Differences between Discrete Timepoints

**Figure 5:**
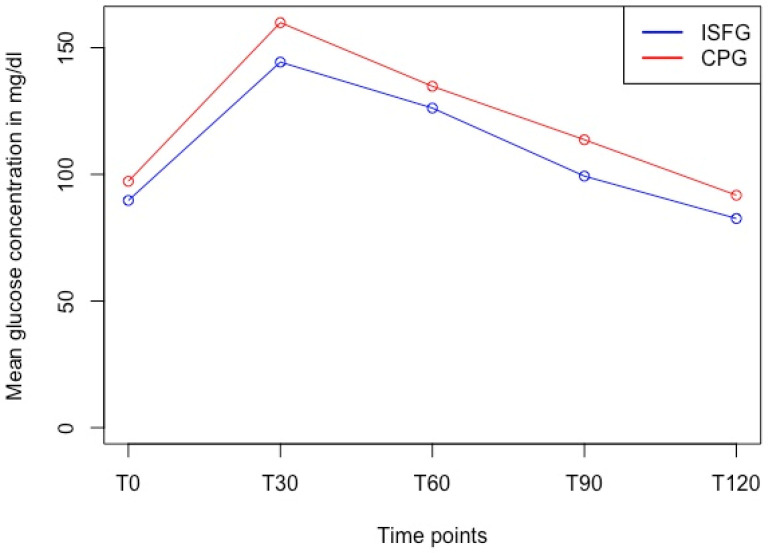
Glucose dynamics of ISFG and CPG in the OGTT over time at discrete timepoints. Paired *t*-test revealed significant differences (*p* < 0.001) at each timepoint with ISFG systematically under-reporting the CPG measurements. Abbreviations: ISFG = interstitial fluid glucose, CPG = capillary blood glucose, T0 = fasting baseline, T30, 60, 90, 120 = 30, 60, 90, 120 min post oral glucose tolerance test.

We used a paired *t*-test to show statistically significant differences between CPG and ISFG at all timepoints (T0: 97.3 mg/dL vs. 89.7 mg/dL, T30: 159.9 mg/dL vs. 144.3 mg/dL, T60: 134.8 mg/dL vs. 126.2 mg/dL, T90: 113.7 mg/dL vs. 99.3 mg/dL, and T120: 91.8 mg/dL vs. 82.6 mg/dL; *p* < 0.001) with moderate to large effect sizes (d = 0.57–1.02) (Table 2) and with ISFG systematically under-reporting the reference system CPG (Figure 5).

## 4. Discussion

In the present study, we assessed the accuracy of CGM-derived ISFG concentrations in comparison to capillary blood glucose (CPG) concentrations using different methods for analytical (MARD, systematic bias) and clinical (e.g., CEG, agreement rates meeting ISO 15197:2013 criteria) point accuracy in a healthy male, normoglycemic study population.

MARD is a method that allows the condensation of the measurement accuracy of the CGM sensors into one single value and can easily be computed [18]. We found that the overall mean absolute relative difference (MARD) was 12.9% (CI: 11.8–14%). Due to its simplicity, MARD is frequently used in the literature [11,19,20]. In one study, the performance of the Freestyle Libre 2 sensor was assessed against plasma venous blood glucose in 144 adults with diabetes over a 14-day wear period [21]. The MARD for the early wear period (days 1 to 3; similar to the wear period in our study) was 10.0%. Conversely, Jin et al. investigated the accuracy of the Freestyle Libre 2 sensor in healthy, non-diabetic females and found an overall MARD of 27.5% [11], highlighting potential discrepancies in sensor performance across different populations. To the knowledge of the authors, these are the only studies that validated the second generation of the Freestyle Libre sensor, offering controversial results. A reason for varying MARDs could be that MARD not only describes the measurement accuracy of the sensor itself, but is also significantly influenced by the protocol in which the sensor performance is assessed [22]. Furthermore, physiological differences between blood and interstitial glucose fluctuations creating a “lag time” between both measurements may have a substantial effect on MARD, especially in cases when glucose changes quickly, as it did in the OGTT [17]. However, previous assessments of the first generation of the Freestyle Libre sensor reported MARDs of 12.3% [19] and 13.2% [20] in type-1 diabetic populations. As another guideline, the typical overall MARD found in other studies with commercially available CGMs ranged from 10–13% [3,17,19,23]. In summary, these findings indicate an acceptable MARD in our study that is in concurrence with the literature.

Regarding the second requirement of the ISO 15197:2013 criteria, 67.7% (n = 149) of the paired glucose data fell inside the predetermined bounds, whereas 32.3% (n = 71) of the data did not satisfy the ISO 15197:2013 requirements. Our findings are congruent with the results stated by Afeef et al., where only 68% of sensor readings satisfied the criteria [4]. Interestingly, a systematic review found that approximately 46% of the CGM systems on the market do not comply with ISO 15197:201323. A reason for that could be that this criterion was initially created to specify the levels of accuracy and precision of glucometer devices, but not especially of CGM sensors.

Furthermore, no uniform methods for the performance accuracy of CGM sensors exist, which leads to varying methods being applied in previous studies [14]. Nevertheless, when examining the clinical point accuracy of CGM-derived data, the CEG analysis showed that >99% of the matched glucose pairs fall into the permitted range and have either no effect on clinical action (zone A) or altered clinical action with little or no effect on clinical outcomes (zone B). These findings are in concurrence with those of previous research [24,25,26] and suggest that therapeutic judgments would not be significantly impacted by CGM values that differ from the reference technique. Both results for the ISO 15197:2013 criteria, taken together, show that the clinical accuracy of the Freestyle Libre 2 sensor is acceptable, but not optimal when it comes to measuring glucose concentrations in the interstitial fluid, especially when deriving implications for clinical decision-making.

The systemic bias analysis can offer good estimators for data point dispersion, as well as location, and is, therefore, a valuable method to assess the accuracy of CGM systems [17]. In our study, we used an OGTT to trigger a glucose fluctuation and evaluate the extent of sensor deviation compared to the reference method. CPG demonstrated a systematic bias of 11.1 mg/dL compared to ISFG, indicating that the sensor readings were systematically lower than those of the finger-prick method. Specifically, the differences between both measurement systems were statistically significant at all timepoints during the OGTT with medium to large effect sizes (d = 0.57–1.02) and with ISFG systematically under-reporting the reference system CPG. A possible reason for systematically lower ISFG values could be physiological differences in the measurement site. After glucose ingestion, glucose first enters the bloodstream from the small intestine before it reaches the interstitial fluid by diffusion down a concentration gradient. Therefore, the finger-prick method measures concentrations before the glucose enters the interstitial fluid and reaches the sensor. A time lag of up to two minutes between the FreeStyle Libre 2 sensor and CPG is reported in Alva et al., especially when glucose fluctuates rapidly [21]. This could explain the systemic under-reporting of the reference system, as the glucose concentration in the interstitial fluid has not yet taken up with the time lag.

The generalizability of the study is limited by the fact that only male participants were recruited for the study. Physiological differences in glucose homeostasis are documented between the sexes, with women tending to have lower fasting glucose and a larger increase from fasting to 2 h post OGTT plasma glucose levels compared to men [27,28]. To eliminate any potential confounding effects of sex on the study results, we limited our participant recruitment to the male sex. Furthermore, another limitation and reason for including only male participants was that the male student population at the UniBw accounts for more than 90% and the data was, therefore, constrained naturally.

In a less controlled environment, such as free-living conditions, the performance of CGM sensors may differ from that observed in clinical or laboratory settings. Factors like physical activity, diet, stress, and varying daily routines can introduce additional variability in glucose levels, potentially affecting sensor accuracy. A study by Moser et al. found that physical activity significantly influences CGM accuracy, as exercise can cause rapid fluctuations in glucose levels that the sensor may not capture accurately [29]. Similarly, another study by Bailey et al. highlighted that environmental factors, such as temperature and humidity, can impact sensor performance, potentially leading to discrepancies between sensor readings and actual blood glucose levels [3].

In free-living conditions, these variables are harder to control, which may result in greater discrepancies between sensor readings and true blood glucose levels. Moreover, research suggests that even CGM systems marketed as ‘calibration-free’ might require more frequent calibration in real-world settings to maintain accuracy [30]. Sensors can result in more comparable glucose readings between ISFG and CBG values and potentially lower MARD if the devices are calibrated beforehand with the reference method of choice. This highlights the importance of considering real-world scenarios when evaluating the effectiveness of CGM systems.

In the future, individuals with diabetes, in addition to healthy participants, should be included in the study. Furthermore, a clustering of events and stratification of glycemic ranges in hypo- (≤70 mg/dL), eu- (72–178 mg/dL), and hyperglycemia (≥180 mg/dL) could shed more light on the dynamics of glucose fluctuations and the performance accuracy of the sensor in these distinct ranges. To better address the continuous nature of glucose dynamics, there exist other promising suggestions for advanced data handling [31] and accuracy assessment [32] for further research.

## 5. Conclusions

Taken together, the results of our study reveal that the accuracy of the FreeStyle Libre 2 sensor in measuring glucose levels is not optimal when compared to values derived from capillary plasma samples in young, healthy men during rapid changes in glucose concentrations. Clinical decision-making should not be solely based on ISFG readings, as CGM data seems to be underestimating CPG values. Phases of hypo- and hyperglycemia can be left unseen because CGM devices might not accurately record blood glucose levels during these events. Still, CGM sensors provide a convenient method to track glucose fluctuations in the everyday lives of healthy adults who are interested in learning and understanding their inherent glucose dynamics.

## Figures and Tables

**Table 1:** Descriptive characteristics of the study population.

Variable	Mean ± SD ^1^	Range
Age (years)	25.5 (±4.5)	20.0–40.0
Height (m)	1.81 (±0.05)	1.73–1.90
Body mass (kg)	84.9 (±13.4)	64.0–122.4
BMI (kg/m^2^)	25.9 (±3.5)	20.5–35.2
Body fat (%)	15.8 (±7.7)	6.5–40.3
Muscle Mass (kg)	34.3 (±4.6)	25.6–46.2

^1^ Abbreviations: SD: standard deviation, BMI: body mass index.

**Table 2:** CPG, ISFG, MARD, 95%-CI, *p*-value, and effect size of the paired t-test clustered for the distinct measurement points (T0, T30, T60, T90, and T120).

Timepoint	CPG ^1^ (mg/dL)	ISFG (mg/dL)	MARD (%)	95%-CI	*p*-Value	Cohen’s d
T0	97.3 ± 8.5	89.7 ± 12.6	12.5	10.9–14.1	<0.001	0.61
T30	159.9 ± 24.6	144.3 ± 29.3	14.2	12.0–16.4	<0.001	0.63
T60	134.8 ± 29.8	126.2 ± 31.0	10.9	9.4–12.5	<0.001	0.57
T90	113.7 ± 21.5	99.3 ± 20.6	14.6	12.5–16.6	<0.001	1.02
T120	91.8 ± 21.2	82.6 ± 18.4	12.3	10.9–13.8	<0.001	0.92

^1^ Abbreviations: CPG = capillary blood glucose, ISFG = interstitial fluid glucose, MARD = mean absolute relative difference, CI = confidence interval, T0 = fasting baseline, T30, 60, 90, 120 = 30, 60, 90, 120 min post oral glucose tolerance test.

## Data Availability

The raw data supporting the conclusions of this article will be made available by the authors on request.
